# Long noncoding RNA *LINC00324* promotes retinoblastoma progression by acting as a competing endogenous RNA for microRNA-769-5p, thereby increasing STAT3 expression

**DOI:** 10.18632/aging.103075

**Published:** 2020-05-05

**Authors:** Yi Dong, Guangming Wan, Panshi Yan, Cheng Qian, Fuzhen Li, Guanghua Peng

**Affiliations:** 1 Department of Ophthalmology, First Affiliated Hospital of Zhengzhou University, Henan Province Eye Hospital, Zhengzhou 450052, Henan, China; 2 School of Basic Medical Sciences, Zhengzhou University, Zhengzhou 450002, Henan, China

**Keywords:** long intergenic non-protein-coding RNA 324, LINC00324, retinoblastoma, microRNA-769-5p, signal transducer and activator of transcription 3

## Abstract

Long intergenic non–protein-coding RNA 324 (*LINC00324*) is abnormally expressed in multiple human cancer types and plays an important role in cancer initiation and progression. This study showed that *LINC00324* was expressed at higher levels in retinoblastoma (RB) tumors and cell lines than in control samples. Increased *LINC00324* expression closely correlated with the TNM stage, optic nerve invasion, and shorter overall survival among patients with RB. The knockdown of *LINC00324* decreased RB cell proliferation, colony formation, migration, and invasion, and promoted apoptosis and cell cycle arrest *in vitro* as well as hindered tumor growth *in vivo*. With respect to the mechanism, *LINC00324* acted as a competing endogenous RNA for microRNA-769-5p (miR-769-5p) in RB cells. The mRNA of signal transducer and activator of transcription 3 (*STAT3*) was identified as a direct target of miR-769-5p in RB cells. Rescue experiments indicated that restoration of STAT3 expression attenuated the tumor-suppressive actions of miR-769-5p in RB cells. Downregulation of miR-769-5p or restoration of STAT3 almost completely reversed the effects of *LINC00324* knockdown on RB cells. Our findings describe a novel RB-related *LINC00324*–miR-769-5p–STAT3 axis that is implicated in the malignancy of RB *in vitro* and *in vivo*. This study may point to innovative therapeutic targets in RB.

## INTRODUCTION

Retinoblastoma (RB) is a human cancer derived from photoreceptor precursor cells [1, 2]. RB is characterized by rapid growth and invasion into the optic nerve and central nervous system [3]. It usually occurs in children aged <5 years, and accounts for 5% of the cases of blindness among children [4]. Multiple therapeutic options, including surgical resection, laser photocoagulation, chemotherapy, radiotherapy, and focal therapy, have undergone significant advancements in the last few decades [5]. Unfortunately, the clinical outcomes of patients with RB are still unsatisfactory, with a death rate of approximately 70% in underdeveloped and moderately developed countries [6]. The existing literature suggests that a variety of factors, such as genetic and epigenetic alterations, are implicated in the initiation and progression of RB [7–9]; however, the pathogenesis of RB is complex, and the molecular events underlying this condition remain largely unknown. Therefore, further investigation into the mechanisms underlying RB formation and progression is important for identifying potential targets for the diagnosis and management of RB.

MicroRNAs (miRNAs, miRs) are a large family of endogenous short noncoding RNA molecules containing approximately 18–25 nucleotides [10]. MiRNAs have the capacity to suppress gene expression by binding (via imperfect or perfect base-pairing) with the 3′-untranslated region (3′-UTR) of their target mRNAs, resulting in either mRNA degradation or translational inhibition [11]. Accumulated evidence has confirmed the crucial regulatory role of miRNAs in diverse physiological and pathological processes, including cell proliferation, cell death, differentiation, metabolism, and even carcinogenesis [12–14]. A number of miRNAs are aberrantly expressed during the genesis and progression of RB [15–17]. Thus, it is urgently necessary to examine the specific functions of miRNAs in RB and to delineate their mechanism of action.

Long noncoding RNAs (lncRNAs) are a group of transcripts that are longer than 200 nucleotides in length [18]. LncRNAs lack any protein coding ability, and yet exert significant effect on the regulation of gene expression at the epigenetic, transcriptional, and post-transcriptional levels through a variety of mechanisms, including interactions with DNAs, RNAs, and proteins [19]. In recent years, lncRNAs have received much attention because of their crucial regulatory roles in the modulation of important biological processes [20, 21]. Recent studies indicate that numerous lncRNAs are dysregulated in RB and affect the aggressive characteristics of RB cells by serving as oncogenes or tumor suppressors [22–24]. Underexpressed miRNAs typically exert tumor-suppressive effects on the progression of RB, whereas overexpressed miRNAs play oncogenic roles [25]. Accordingly, some lncRNAs may serve as attractive biomarkers of (and/or therapeutic targets in) RB.

Long intergenic non–protein-coding RNA 324 (*LINC00324*) is abnormally expressed in multiple human cancer types [26–28] and performs important functions in cancer initiation and progression. Nevertheless, the expression profile, clinical relevance, specific effects, and regulatory mechanisms underlying the action of *LINC00324* in RB have not been fully elucidated. Therefore, this study was aimed at measuring the expression of *LINC00324* in RB cells and determining its clinical value in patients with RB. Furthermore, the effects of a *LINC00324* knockdown on the malignant characteristics of RB cells *in vitro* and *in vivo* were explored in a series of functional experiments. Moreover, the molecular mechanisms via which *LINC00324* regulates RB progression were comprehensively investigated.

MiR-769-5p is weakly expressed in non–small cell lung [29] and colorectal cancers [30]; however, miR-769-5p is highly expressed in melanoma [31]. STAT3 is a key transcription factor belonging to the STAT family and can be activated by a variety of cytokines, growth factors, and interferons [32]. It is overexpressed in RB and promotes the aggressiveness of RB in vitro and in vivo [33–36]. Here, our results clearly demonstrated that *LINC00324* performed cancer-promoting actions through regulating the miR-769-5p/STAT3.

## RESULTS

### *LINC00324* is upregulated in RB tissues and cell lines

**Figure 1:**
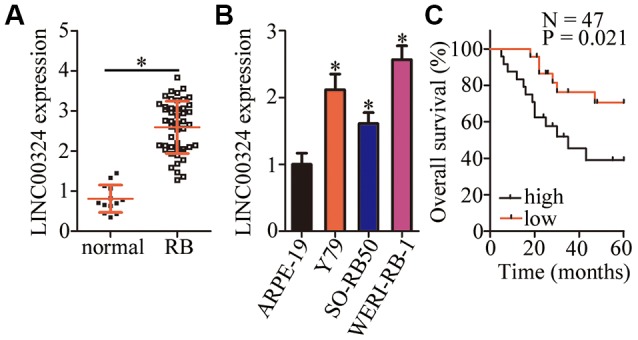
***LINC00324* expression is high in retinoblastoma (RB) tumors and cell lines.** (**A**) The expression of *LINC00324* was determined in 47 RB tissue samples and 13 normal retinal tissue samples by RT-qPCR. *P < 0.05 vs. normal retinal tissue samples. (**B**) *LINC00324* expression in three RB cell lines (Y79, SO-RB50, and WERI-RB-1) and in a normal retinal pigmented epithelial cell line, ARPE-19, was assessed via RT-qPCR. *P < 0.05 vs. ARPE-19 cells. (**C**) The relationship between *LINC00324* expression and overall survival in the 47 patients with RB was evaluated via the Kaplan–Meier survival curve and log rank test. *P* = 0.021.

To study the specific functions of *LINC00324* in RB, we first quantified the expression of this lncRNA in 47 RB tissue samples and 13 normal retinal tissue samples. The results of reverse-transcription quantitative polymerase chain reaction (RT-qPCR) made it clear that *LINC00324* was overexpressed in RB tissue samples relative to that in normal retinal tissues (Figure 1A, *P* < 0.05). We also determined *LINC00324* expression in three RB cell lines (Y79, SO-RB50, and WERI-RB-1) and in a normal retinal pigmented epithelial cell line, ARPE-19. The expression of *LINC00324* was markedly higher in all three RB cell lines compared with that in ARPE-19 cells (Figure 1B, *P* < 0.05).

To examine the relationship between *LINC00324* expression and clinical parameters among the patients with RB, the participants were assigned to either the low–*LINC00324* expression group or high–*LINC00324* expression group based on the median level of *LINC00324* in the RB tumors. The χ^2^ test revealed that high *LINC00324* expression correlated with the TNM stage (*P* = 0.039) and optic nerve invasion (*P* = 0.041; Table 1). Of note, patients with RB expressing high levels of *LINC00324* demonstrated worse overall survival as compared with the patients with low *LINC00324* expression (Figure 1C, *P* = 0.021). Based on these results, we speculate that *LINC00324* may play a crucial role in the malignancy of RB.

**Table 1:** Correlation between *LINC00324* and clinical parameters in patients with RB (n = 47).

**Parameters**	***LINC00324* expression**		**P**
	**High (n=24)**	**Low(n=23)**	
**Sex**			0.147
Male	10	15	
Female	14	8	
**Age**			0.193
< 5 years	15	19	
≥ 5 years	9	4	
**Enucleated tumor location**			0.772
Right	12	10	
Left	12	13	
**Differentiation grade**			0.752
Well/moderate	16	17	
Poor/undifferentiated	8	6	
**TNM stage**			0.039^a^
I+II	6	13	
III+IV	18	10	
**Optic nerve invasion**			0.041^a^
Negative	9	16	
Positive	15	7	

^a^*P* < 0.05 (chi-square test).

### Depletion of *LINC00324* inhibits the malignant characteristics of RB cells *in vitro*

**Figure 2:**
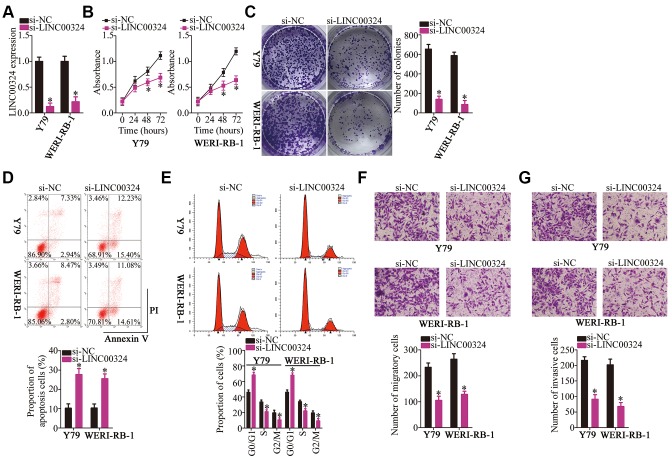
***LINC00324* knockdown inhibits Y79 and WERI-RB-1 cell proliferation, colony formation, migration, and invasion, and promotes apoptosis *in vitro.*** (**A**) Either si-LINC00324 or si-NC was transfected into Y79 and WERI-RB-1 cells. The transfected cells were collected 48 h later and used for evaluation of transfection efficiency. *P < 0.05 vs. the si-NC group. (**B**, **C**) The proliferative and colony-forming abilities of *LINC00324*-depleted Y79 and WERI-RB-1 cells were examined using CCK-8 and colony formation assays, respectively. *P < 0.05 vs. group si-NC. (**D**, **E**) Apoptosis and cell cycle was analyzed by flow cytometry in Y79 and WERI-RB-1 cells transfected with either si-LINC00324 or si-NC. *P < 0.05 vs. group si-NC. (**F**, **G**) Transwell migration and invasion assays were performed to assess the migratory and invasive abilities of Y79 and WERI-RB-1 cells after transfection with either si-LINC00324 or si-NC. *P < 0.05 vs. the si-NC group.

The cell lines Y79 and WERI-RB-1, which demonstrated the highest expression of *LINC00324* among the three RB cell lines, were selected for the subsequent experiments, and were transfected with either small interfering RNA (siRNA) targeting *LINC00324* (si-LINC00324) or a negative control siRNA (si-NC). The levels of *LINC00324* reduced significantly in Y79 and WERI-RB-1 cells after treatment with *si-LINC00324*, as evidenced by RT-qPCR (Figure 2A, *P* < 0.05). A Cell Counting Kit-8 (CCK-8) assay was used to investigate the effect of *LINC00324* downregulation on the proliferation of RB cells. Transfection with si-LINC00324 clearly decreased the proliferative ability of Y79 and WERI-RB-1 cells (Figure 2B, *P* < 0.05). Consistent with this result, a colony formation assay indicated that *LINC00324* knockdown significantly decreased the colony-forming ability of Y79 and WERI-RB-1 cells (Figure 2C, *P* < 0.05).

Apoptosis induction and cell cycle arrest helps to suppress the proliferation of tumor cells. Accordingly, flow-cytometric analysis was performed to test whether *LINC00324* knockdown affects the apoptosis and cell cycle status of RB cells. Interference with *LINC00324* expression promoted the apoptosis (Figure 2D, P < 0.05) and G0–G1 cell cycle arrest (Figure 2E, P < 0.05) of Y79 and WERI-RB-1 cells. Hence, the suppression of RB cell proliferation by *LINC00324* knockdown could be attributed to the induction of apoptosis and cell cycle arrest. Furthermore, Transwell migration and invasion assays were conducted to assess the effects of the *LINC00324* knockdown on the migration and invasiveness of RB cells. Microscopy images showed that the knockdown of *LINC00324* strongly inhibited the migratory (Figure 2F, *P* < 0.05) and invasive (Figure 2G, *P* < 0.05) abilities of Y79 and WERI-RB-1 cells relative to those in the si-NC groups. Taken together, these results suggest that *LINC00324* may promote the progression of RB.

### *LINC00324* functions as a competing endogenous RNA (ceRNA) of miR-769-5p in RB cells

**Figure 3:**
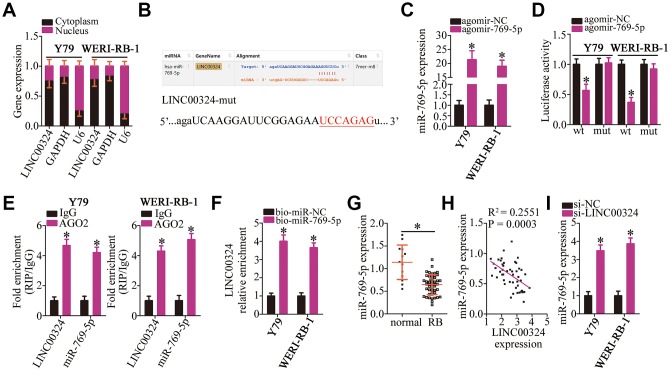
***LINC00324* acts as a sponge on miR-769-5p in RB cells.** (**A**) RNA was extracted from cytoplasmic and nuclear fractions, and then subjected to RT-qPCR to characterize the distribution of *LINC00324* inside Y79 and WERI-RB-1 cells. (**B**) The miR-769-5p–binding sequences in *LINC00324* were predicted using starBase 3.0. The designed mutant binding site is also shown. (**C**) Either agomir-769-5p or agomir-NC was transfected into Y79 and WERI-RB-1 cells. RT-qPCR was conducted at 48 h post-transfection to quantitate miR-769-5p expression. *P < 0.05 vs. group agomir-NC. (**D**) Luciferase activity was measured using the luciferase reporter assay in Y79 and WERI-RB-1 cells cotransfected with either LINC00324-wt or LINC00324-mut and either agomir-769-5p or agomir-NC. *P < 0.05 vs. the agomir-NC group. (**E**) The interaction between *LINC00324* and miR-769-5p in Y79 and WERI-RB-1 cells was detected via the RIP assay. *LINC00324* and miR-769-5p expression was measured by RT-qPCR. *P < 0.05 vs. the IgG group. (**F**) Y79 and WERI-RB-1 cells were transfected with bio-miR-769-5p or bio-miR-NC and their lysates were incubated with streptavidin-coupled beads to form bio-miRNA-lncRNA complexes. The *LINC00324* enrichment was analyzed by means of RT-qPCR analysis. *P < 0.05 vs. the bio-miR-NC group. (**G**) The relative expression of miR-769-5p in 47 RB tissue samples and 13 normal retinal tissue samples was determined using RT-qPCR, and was normalized to that of U6. *P < 0.05 vs. normal retinal tissue samples. (**H**) The correlation between the expression of *LINC00324* and miR-769-5p was investigated by Spearman’s correlation analysis; R^2^ = 0.2551, *P* = 0.0003. (**I**) After transfection with either si-LINC00324 or si-NC, the expression of miR-769-5p in Y79 and WERI-RB-1 cells was determined via RT-qPCR. *P < 0.05 vs. the si-NC group.

Growing evidence suggests that lncRNAs can competitively bind to certain miRNAs and relieve the repressive actions of these miRNAs on the expression of their target genes [37]. To illustrate the mechanisms underlying the oncogenic activities of *LINC00324* in RB cells, we first determined the localization of *LINC00324*. As presented in Figure 3A, *LINC00324* was mainly located in the cytoplasm of Y79 and WERI-RB-1 cells, indicating that *LINC00324* may act as a ceRNA for certain miRNAs in RB cells. We therefore tried to predict whether the miRNAs could interact with *LINC00324* using starBase 3.0. MiR-769-5p contains sequences that are complementary to *LINC00324*; the predicted site for miR-769-5p binding in *LINC00324* has been depicted in Figure 3B. MiR-769-5p has been reported to be downregulated and to function as a tumor-suppressive miRNA in multiple human cancer types [29, 30].

To verify the interaction between *LINC00324* and miR-769-5p, a luciferase reporter assay was performed in Y79 and WERI-RB-1 cells after cotransfecting them with either miR-769-5p agomir (agomir-769-5p) or agomir-NC and either reporter plasmid LINC00324-wt (encoding the wild-type miR-769-5p–binding site) or LINC00324-mut (encoding a mutated miR-769-5p–binding site). Transfection with agomir-769-5p greatly improved the expression of miR-769-5p (Figure 3C, *P* < 0.05) and decreased the luciferase activity of LINC00324-wt in Y79 and WERI-RB-1 cells (Figure 3D, *P* < 0.05). In contrast, no inhibitory effect on the luciferase activity of LINC00324-mut was observed, suggesting that in RB cells, miR-769-5p can bind directly to *LINC00324* via its binding site. RNA immunoprecipitation (RIP) and RNA pull-down assays were then performed to confirm the direct binding between *LINC00324* and miR-769-5p in RB cells. The results of RIP assay revealed that *LINC00324* and miR-769-5p were specifically enriched in the AGO2 immunoprecipitate as compared with that in the IgG control group (Figure 3E, *P* < 0.05). For the RNA pull-down assay, Y79 and WERI-RB-1 cells were transfected with bio-miR-miR-769-5p or bio-miR-NC. The lysates were incubated with streptavidin-coupled beads to form bio-miRNA-lncRNA complexes. The data displayed that LINC00324 enrichment was higher in the bio-miR-769-5p group in comparison with that in bio-miR-NC group (Figure 3F, *P* < 0.05)

After that, an RT-qPCR assay was performed to quantify miR-769-5p expression in 47 RB tissue samples and 13 normal retinal tissue samples, which demonstrated significant downregulation of miR-769-5p in RB tumors relative to that in normal retinal tissue (Figure 3G, *P* < 0.05). Of note, a negative correlation between the expression of *LINC00324* and miR-769-5p levels was identified in the 47 RB tissue samples by Spearman’s correlation analysis (Figure 3H; R^2^ = 0.2551, *P* = 0.0003). Additionally, the effect of *LINC00324* knockdown on miR-769-5p expression was tested by RT-qPCR. The results demonstrated that transfection with si-LINC00324 markedly increased the expression of miR-769-5p in Y79 and WERI-RB-1 cells (Figure 3I, *P* < 0.05). Altogether, the above data led to the conclusion that *LINC00324* may act as an miR-769-5p sponge in RB cells.

### MiR-769-5p serves as a tumor-suppressive miRNA in RB cells

**Figure 4:**
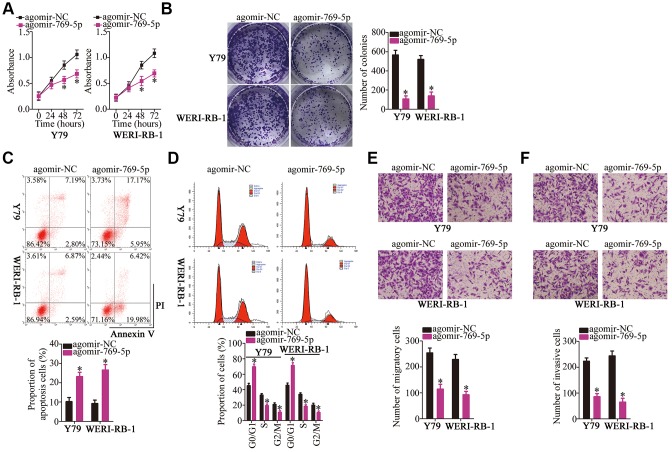
**Forced expression of miR-769-5p attenuates the growth and metastasis of Y79 and WERI-RB-1 cells *in vitro.*** (**A**, **B**) Y79 and WERI-RB-1 cells were transfected with either agomir-769-5p or agomir-NC. After transfection, CCK-8 and colony formation assays were performed to assess the effects of miR-769-5p overexpression on the proliferative and colony-forming abilities of RB cells. *P < 0.05 vs. the agomir-NC group. (**C**, **D**) Apoptosis and cell cycle was assessed via flow-cytometric analysis in miR-769-5p–overexpressing Y79 and WERI-RB-1 cells. *P < 0.05 vs. group agomir-NC. (**E**, **F**) Y79 and WERI-RB-1 cells were treated as mentioned above, and Transwell migration and invasion assays were performed to assess cell migration and invasion. *P < 0.05 vs. group agomir-NC.

To further investigate the association of miR-769-5p with the malignant characteristics of RB cells, agomir-769-5p was transfected into Y79 and WERI-RB-1 cells to increase the expression of endogenous miR-769-5p. Transfection with agomir-NC served as the control. Then, a CCK-8 assay, colony formation assay, and flow cytometry were carried out to determine cell proliferation, colony formation, and apoptosis status, respectively, of miR-769-5p–overexpressing Y79 and WERI-RB-1 cells. Ectopic expression of miR-769-5p clearly inhibited the proliferation and colony formation ability of Y79 and WERI-RB-1 cell proliferation (Figure 4A, 4B *P* < 0.05), and promoted apoptosis (Figure 4C, *P* < 0.05) and G0–G1 cell cycle arrest (Figure 4D, *P* < 0.05). In addition, the overexpression of miR-769-5p decreased the migration (Figure 4E, *P* < 0.05) and invasiveness (Figure 4F, *P* < 0.05) of Y79 and WERI-RB-1 cells, as determined by the Transwell migration and invasion assays. Collectively, these findings demonstrate that miR-769-5p inhibited the malignant phenotype of RB cells *in vitro*.

### *STAT3* mRNA is a direct target of miR-769-5p in RB cells

**Figure 5:**
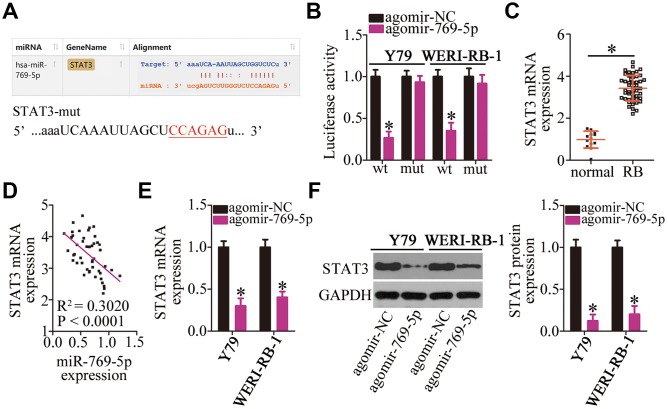
**Validation of *STAT3* mRNA as a direct target of miR-769-5p in RB cells.** (**A**) The wild-type miR-769-5p–binding site in the 3**′**-UTR of *STAT3* mRNA was predicted by bioinformatic analysis. The mutant binding site is also shown. (**B**) The luciferase reporter plasmid harboring either the wild-type or mutant miR-769-5p–binding site was cotransfected with either agomir-769-5p or agomir-NC into Y79 and WERI-RB-1 cells. The Dual-Luciferase Reporter Assay System was used to detect the Firefly luciferase activity by normalizing it to the activity of *Renilla* luciferase. *P < 0.05 vs. group agomir-NC. (**C**) RT-qPCR was carried out to quantitate *STAT3* mRNA in the 47 RB tissue samples and 13 normal retinal tissue samples. *P < 0.05 vs. normal retinal tissue samples. (**D**) Spearman’s correlation analysis was performed to study the correlation between the expression of miR-769-5p and *STAT3* mRNA among the 47 RB tissue samples; R^2^ = 0.3020, *P* < 0.0001. (**E**, **F**) Y79 and WERI-RB-1 cells were transfected with either agomir-769-5p or agomir-NC. The mRNA and protein levels of STAT3 were measured by RT-qPCR and western blotting, respectively. *P < 0.05 vs. the agomir-NC group.

To illustrate the potential mechanisms of action of miR-769-5p in RB cells, bioinformatic analysis was carried out to search for the potential targets of miR-769-5p. This analysis showed that *STAT3* mRNA possesses a conserved miR-769-5p–binding site (Figure 5A). A luciferase reporter assay was performed to investigate this bioinformatic prediction. The results indicated that transfection with agomir-769-5p significantly reduced the luciferase activity of the reporter plasmid containing the wild-type *STAT3*-binding site in both Y79 and WERI-RB-1 cells (*P* < 0.05). By contrast, miR-769-5p overexpression did not affect the luciferase activity of the reporter plasmid harboring mutations in the miR-769-5p–binding sequences (Figure 5B). Next, we measured the expression of *STAT3* in the 47 RB tissue samples and 13 normal retinal tissue samples. The findings of RT-qPCR analyses demonstrated that *STAT3* mRNA was significantly upregulated in the RB tissue samples compared with that in the normal retinal tissue samples (Figure 5C, *P* < 0.05). Additionally, the mRNA level of *STAT3* was found to be negatively correlated with the miR-769-5p levels in RB tumors (Figure 5D; R^2^ = 0.3020, *P* < 0.0001). RT-qPCR and western blotting were carried out to test whether the mRNA and protein levels of STAT3 could be downregulated by miR-769-5p in RB cells. Resumption of miR-769-5p expression significantly decreased *STAT3* mRNA (Figure 5E, *P* < 0.05) and protein (Figure 5F, *P* < 0.05) expression in Y79 and WERI-RB-1 cells. These results confirmed *STAT3* as a direct target gene of miR-769-5p in RB cells.

### Restoring STAT3 expression neutralizes the effects of miR-769-5p upregulation on the malignant phenotype of RB cells

**Figure 6:**
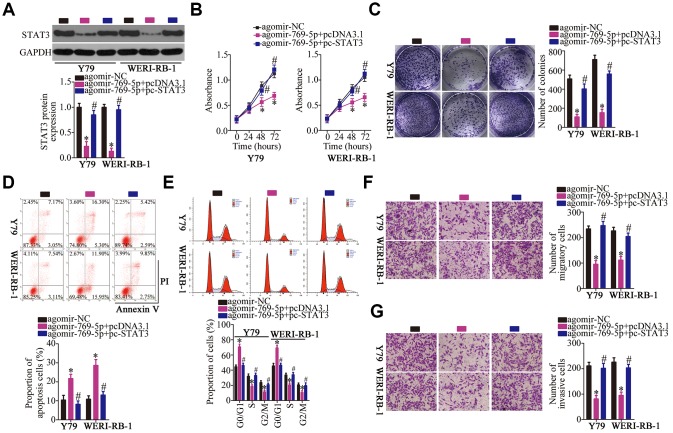
**Reintroduction of STAT3 abrogated the effect of miR-769-5p overexpression on the malignant characteristics of Y79 and WERI-RB-1 cells.** (**A**) Either the STAT3-overexpressing plasmid pc-STAT3 or the empty pcDNA3.1 vector along with agomir-769-5p were transfected into Y79 and WERI-RB-1 cells. The expression of STAT3 protein was measured via western blotting. *P < 0.05 vs. the agomir-NC group, ^#^*P* < 0.05 vs. group agomir-769-5p+pcDNA3.1. (**B**, **C**) The proliferative and colony-forming abilities of the above-mentioned cells were examined using the CCK-8 and colony formation assays, respectively. *P < 0.05 vs. the agomir-NC group, ^#^*P* < 0.05 vs. group agomir-769-5p+pcDNA3.1. (**D**, **E**) Flow-cytometric analysis was performed to investigate the apoptosis and cell cycle statues of Y79 and WERI-RB-1 cells after cotransfection with agomir-769-5p and either pc-STAT3 or pcDNA3.1. *P < 0.05 vs. the agomir-NC group, ^#^*P* < 0.05 vs. group agomir-769-5p+pcDNA3.1. (**F**, **G**) Transwell migration and invasion assays were carried out to assess the migratory and invasive abilities of Y79 and WERI-RB-1 cells treated as described above. *P < 0.05 vs. the agomir-NC group, ^#^*P* < 0.05 vs. group agomir-769-5p+pcDNA3.1.

Having identified *STAT3* as a direct target of miR-769-5p, we next assessed the possibility that STAT3 downregulation may be responsible for the tumor-suppressive roles of miR-769-5p in RB cells. To this end, the STAT3-overexpressing plasmid pc-STAT3 was transfected into the miR-769-5p–overexpressing Y79 and WERI-RB-1 cells. MiR-769-5p upregulation significantly suppressed STAT3 protein expression in Y79 and WERI-RB-1 cells; however, cotransfection with pc-STAT3 almost completely reversed the repression of STAT3 protein expression that was observed upon overexpression of miR-769-5p (Figure 6A, *P* < 0.05). Functional experiments revealed that the restoration of STAT3 expression abrogated the influence of miR-769-5p overexpression on the proliferation (Figure 6B, *P* < 0.05), colony formation (Figure 6C, *P* < 0.05), apoptosis (Figure 6D, *P* < 0.05), cell cycle statues (Figure 6E, *P* < 0.05), migration (Figure 6F, *P* < 0.05), and invasiveness (Figure 6G, *P* < 0.05) of Y79 and WERI-RB-1 cells. Collectively, these data suggest that miR-769-5p attenuated the growth and metastasis of RB cells *in vitro,* at least partly, by downregulating STAT3.

### *LINC00324* knockdown inhibits the malignant behavior of RB cells *in vitro* by acting on the miR-769-5p–STAT3 axis

**Figure 7:**
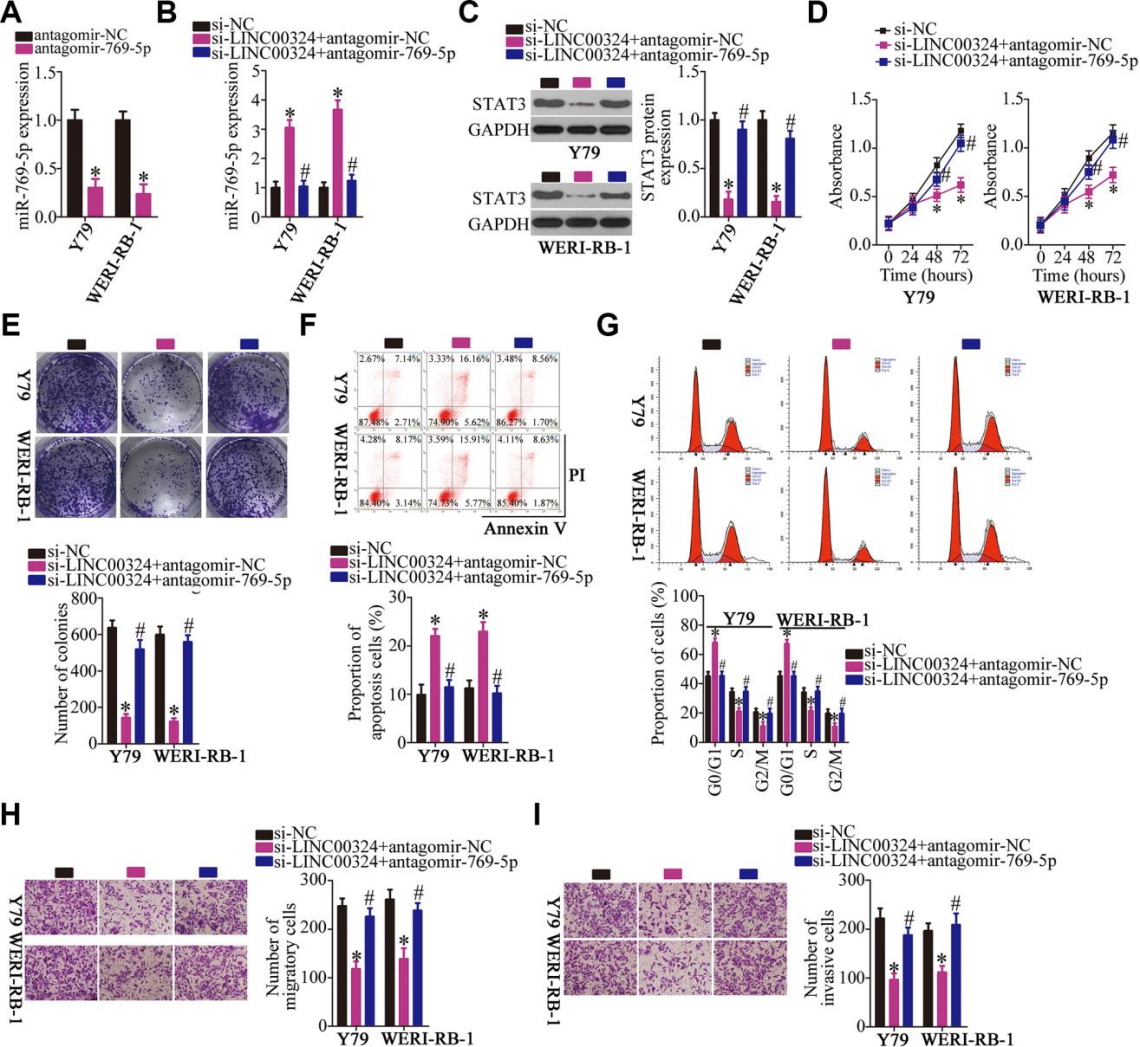
**Downregulation of miR-769-5p neutralizes the effects of *LINC00324* knockdown on RB cells.** (**A**) RT-qPCR analysis was used to quantify the expression of miR-769-5p in Y79 and WERI-RB-1 cells that were transfected with either antagomir-769-5p or antagomir-NC, *P < 0.05 vs. the antagomir-NC group. (**B**, **C**) Si-LINC00324 was cotransfected with either antagomir-769-5p or antagomir-NC into Y79 and WERI-RB-1 cells. After transfection, RT-qPCR and western blotting were performed to measure the expression of miR-769-5p and STAT3 protein, respectively. *P < 0.05 vs. the si-NC group, ^#^*P* < 0.05 vs. group si-LINC00324+antagomir-NC. (**D**–**I**) A CCK-8 assay, colony formation assay, flow-cytometric analysis, and Transwell migration and invasion assays were conducted to analyze the proliferation, colony formation, apoptosis, cell cycle, migration, and invasiveness of the aforementioned cells. *P < 0.05 vs. the si-NC group, ^#^*P* < 0.05 vs. group si-LINC00324+antagomir-NC.

To further test the association between *LINC00324*, miR-769-5p, and STAT3 in RB, a series of rescue experiments were conducted via transfection of si-LINC00324 with either antagomir-769-5p or antagomir-NC into Y79 and WERI-RB-1 cells. First, the efficiency of transfection of antagomir-769-5p was assessed by RT-qPCR. MiR-769-5p was found to be successfully silenced in the antagomir-769-5p–transfected Y79 and WERI-RB-1 cells compared with that in cells transfected with antagomir-NC (Figure 7A, *P* < 0.05). Next, miR-769-5p and STAT3 protein expression in Y79 and WERI-RB-1 cells treated as described above was determined by RT-qPCR and western blotting, respectively. Depletion of *LINC00324* increased the levels of miR-769-5p (Figure 7B, *P* < 0.05) and lowered the expression of STAT3 protein(Figure 7C, *P* < 0.05) in Y79 and WERI-RB-1 cells, whereas cotransfection with antagomir-769-5p abrogated these phenomena. Furthermore, the effects of *LINC00324* knockdown on the proliferation (Figure 7D, *P* < 0.05), colony formation capacity (Figure 7E, *P* < 0.05), apoptosis (Figure 7F, *P* < 0.05), cell cycle statues (Figure 7G, *P* < 0.05), migration (Figure 7H, *P* < 0.05), and invasiveness (Figure 7I, *P* < 0.05) of Y79 and WERI-RB-1 cells were almost completely reversed after cotransfection with antagomir-769-5p.

**Figure 8:**
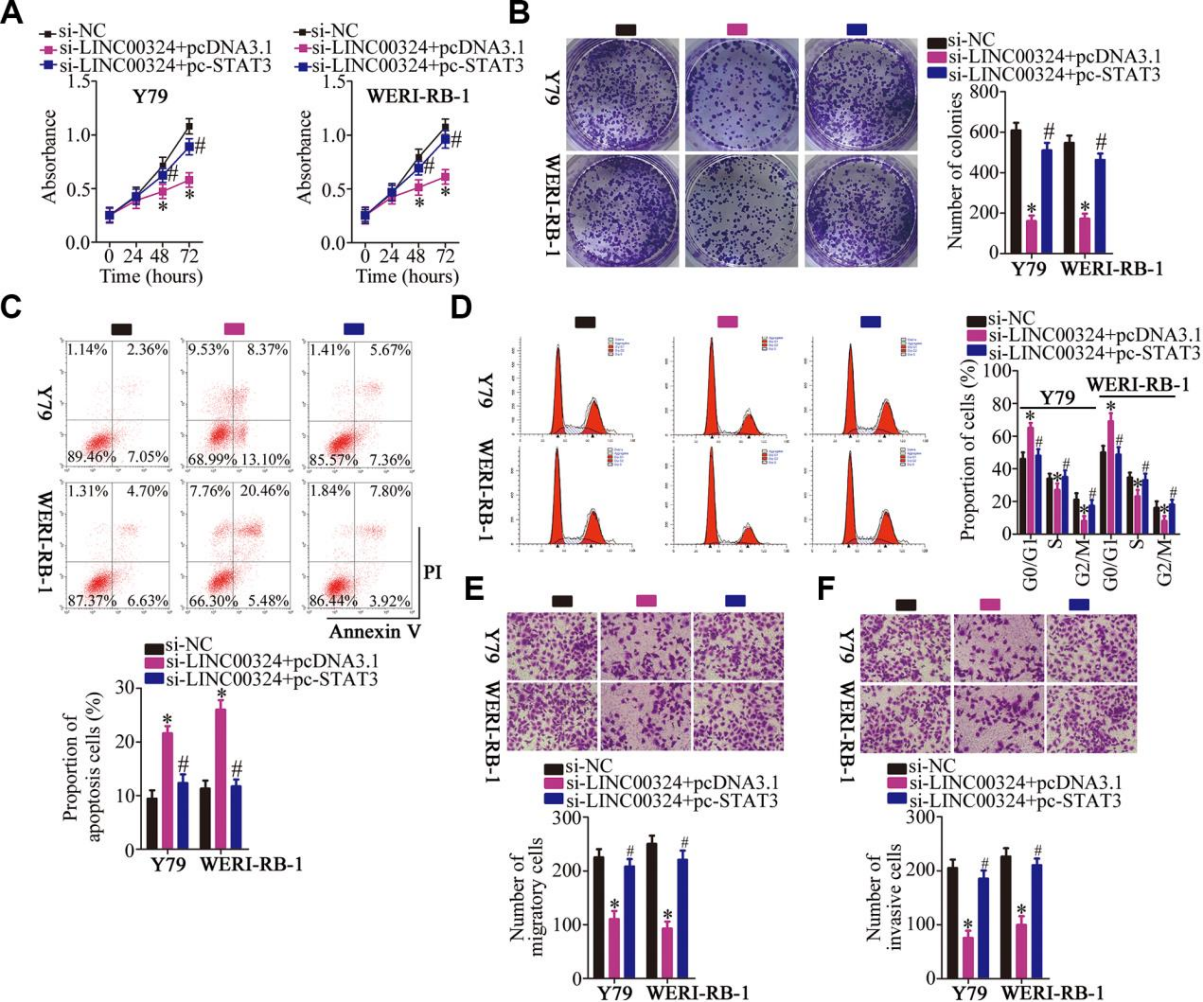
**Reintroduction of STAT3 abolishes the actions of *LINC00324* knockdown on RB cells.** (**A**–**F**) Y79 and WERI-RB-1 cells were cotransfected with si-LINC00324 and pcDNA3.1 or pc-STAT3. The proliferation, colony formation, apoptosis, cell cycle, migration and invasion was respectively determined by CCK-8 assay, colony formation assay, flow-cytometric analysis, and Transwell migration and invasion assays. *P < 0.05 vs. the si-NC group, ^#^*P* < 0.05 vs. group si-LINC00324+ pcDNA3.1.

What's more, rescue experiments were designed and conducted to further prove that whether STAT3 mediates the oncogenic actions of *LINC00324* in RB cells. To this end, pcDNA3.1 or pc-STAT3 alongside si-LINC00324 was cotransfected into Y79 and WERI-RB-1 cells. *LINC00324* silencing strikingly suppressed Y79 and WERI-RB-1 cell proliferation (Figure 8A), colony formation (Figure 8B) and promoted cell apoptosis (Figure 8C) and cell cycle arrest (Figure 8D), while pc-STAT3 cotransfection abrogated these influences. Also, reintroduction of STAT3 attenuated *LINC00324* knockdown induced suppression on Y79 and WERI-RB-1 cell migration (Figure 8E) and invasion (Figure 8F). In short, these findings demonstrate that the *LINC00324*–miR-769-5p–STAT3 axis can regulate the behavior of RB cells *in vitro*.

### *LINC00324* downregulation suppresses the tumor growth of RB cells *in vivo*

**Figure 9:**
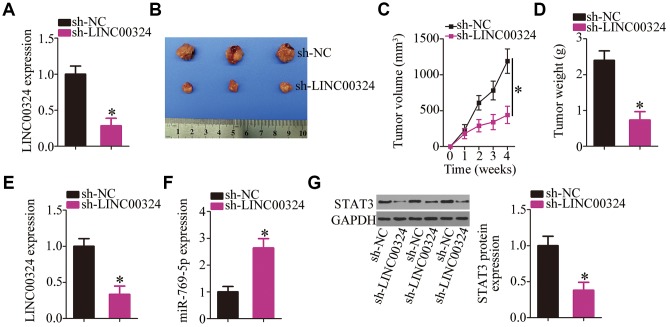
**Depletion of *LINC00324* attenuates the tumor growth of RB cells *in vivo.*** (**A**) RT-qPCR analysis was utilized to assess the efficiency of sh-LINC00324 in Y79 cells. *P < 0.05 vs. the sh-NC. (**B**) The tumor growth rate was significantly suppressed by sh-LINC00324, as compared with that of the sh-NC group. *P < 0.05 vs. the sh-NC group. (**C**) At the conclusion of the assay, the mice were euthanized, and the tumor xenografts were excised. Representative images of tumor xenografts from the sh-LINC00324 and sh-NC groups. (**D**) The average weight of the tumor xenografts was determined in the sh-LINC00324 and sh-NC groups. *P < 0.05 vs. the sh-NC group. (**E**, **F**) RT-qPCR was carried out to analyze the expression of *LINC00324* and miR-769-5p in the tumor xenografts. *P < 0.05 vs. group sh-NC. (**G**) Total protein was isolated from tumor xenografts, and then subjected to western blotting to measure STAT3 protein expression. *P < 0.05 vs. group sh-NC.

Finally, a tumor xenograft model was set up to examine the impact of *LINC00324* knockdown on the tumor growth of RB cells *in vivo*. The efficiency of sh-LINC00324 was determined by RT-qPCR (Figure 9A; *P* < 0.05). Nude mice were injected with Y79 cells stably expressing sh-LINC00324 or sh-NC. The tumor volumes (Figures 9B and C; *P* < 0.05) were markedly smaller in the sh-LINC00324 group compared with that in the sh-NC group. After four weeks, all the mice were euthanized, and the tumor xenografts were excised and weighed. The average weight of tumor xenografts harboring sh-LINC00324 was significantly lower compared with that in the sh-NC group (Figure 9D, *P* < 0.05). Furthermore, RT-qPCR analysis was performed on xenograft tissues. The results showed that the tumor xenografts derived from sh-LINC00324–transfected Y79 cells contained lower amounts of *LINC00324* (Figure 9E, *P* < 0.05) and higher levels of miR-769-5p (Figure 9F, *P* < 0.05) compared with that in the sh-NC group. In addition, lower STAT3 protein expression was noted in the sh-LINC00324 group compared with that in the sh-NC group (Figure 9G, *P* < 0.05). Overall, the above data indicate that the tumor growth of RB cells *in vivo* was inhibited by the *LINC00324* knockdown, and that this phenomenon was possibly mediated by the miR-769-5p–STAT3 axis.

## DISCUSSION

Over the last several decades, an increasing body of evidence has revealed the crucial role of lncRNAs in human diseases, especially cancers [38–40]. In RB, in particular, a variety of lncRNAs have been shown to be aberrantly expressed, and their anomalous expression has been implicated in the malignancy of RB, as these lncRNAs regulate several biological characteristics of tumors [41–43]. Therefore, a greater understanding of the activities of cancer-related lncRNAs in RB may reveal potential novel targets for the diagnosis, prevention, and treatment of this malignant tumor. In this study, we hypothesized that one of the cancer-related lncRNAs, *LINC00324*, may be involved in the progression of RB. To test this hypothesis, we first quantified *LINC00324* expression in RB tumors and cell lines. The clinical significance of *LINC00324* in patients with RB was also examined in detail. In addition, we investigated the specific functions of *LINC00324* with respect to RB progression, and elucidated the mechanism of action of *LINC00324* in RB.

*LINC00324* is upregulated in gastric cancer, and its upregulation significantly correlates with the TNM stage, tumor size, and lymph node metastasis [26]. Patients with gastric cancer featuring *LINC00324* overexpression show shorter overall survival and disease-free survival compared with those with low *LINC00324* expression [26]. In addition, expression of *LINC00324* has been validated as an independent predictive factor of overall survival and disease-free survival among patients with gastric cancer [26]. *LINC00324* is also overexpressed in lung adenocarcinoma [27] and osteosarcoma [28]. Overexpression of *LINC00324* is closely associated with tumor size, distant metastasis, TNM stage, differentiation degree, and poor prognosis among patients with osteosarcoma [28]. Nevertheless, the expression status of *LINC00324* in RB has not been well understood until now. In this study, we found that *LINC00324* is overexpressed in both RB tumors and cell lines. *LINC00324* overexpression in RB tissue samples was significantly correlated with TNM stage and optic nerve invasion. Of note, patients with RB exhibiting *LINC00324* overexpression showed shorter overall survival relative to that in patients with underexpression of *LINC00324*. These observations suggest that *LINC00324* might be a promising biomarker for the diagnosis and/or prognosis of RB.

*LINC00324* exerts oncogenic actions during tumorigenesis and tumor progression. For instance, *LINC00324* overexpression enhances cell proliferation, migration, and invasion in gastric-cancer and inhibits apoptosis *in vitro;* further, it also facilitates tumor growth *in vivo* [26]. The oncogenic activities of *LINC00324* in gastric cancer cells are mediated by its binding to HuR and stabilization of FAM83B expression [26]. In lung adenocarcinoma, *LINC00324* knockdown restricts tumor cell growth and metastasis via regulation of the miR-615-5p–AKT1 axis [27]. In osteosarcoma, *LINC00324* overexpression increases WDR66 expression, thereby promoting tumor cell proliferation and migration *in vitro* [28].

The involvement of *LINC00324* in RB progression has remained elusive. Herein, results from functional experiments demonstrate that siRNA-mediated knockdown of *LINC00324* decreased RB cell proliferation, colony formation, migration, and invasion, and promoted cell apoptosis *in vitro* and inhibited tumor growth *in vivo*. In the determination of cell cycle status, we found that knockdown of LINC00324 induced RB cell apoptosis, including late apoptotic cells and a large group of early apoptotic cells (Figure 2D). In Figure 7F, co-transfection with antagomir-769-5p was performed to rescue the effects of si-LINC00324 on cell apoptosis. However, the effects of si-LINC00324 were not consistent with those in Figure 2E, most of the Annexin V positive cells in "si-LINC00324+antagomir-NC" groups were late apoptotic. This difference may be due to the differences in cell numbers and the impact of cotransfection on the cell cycle status.

We also studied the regulatory mechanisms by which *LINC00324* affects the malignant characteristics of RB cells *in vitro* and *in vivo*. Accumulated evidence suggests that lncRNAs perform their tumor-suppressive or oncogenic functions by interacting with certain miRNAs, and by attenuating the suppressive action of these miRNAs on the expression of their target genes [44–46]. Overall, the results of our study show that *LINC00324* plays an oncogenic role in RB cells by functioning as a ceRNA for miR-769-5p, thereby upregulating STAT3. MiR-769-5p has been found to be downregulated in non–small cell lung cancer [29] and colorectal cancer [30], and it has been found to function as a tumor suppressor. In contrast, miR-769-5p is overexpressed in melanoma [31] and exerts oncogenic effects. These conflicting findings led us to characterize the expression status and roles of miR-769-5p in RB cells. Our results show for the first time that miR-769-5p is downregulated in RB. Overexpressed miR-769-5p directly targeted *STAT3* mRNA, thereby reducing the aggressiveness of RB cells.

STAT3 is overexpressed in RB and enhances the malignancy of RB by increasing cellular proliferation, colony formation, migration, invasion, and tumorigenesis while decreasing apoptosis [33–36]. The results of the present study illustrate a novel upstream mechanism that regulates the expression of STAT3 in RB. *LINC00324*—which contains miR-769-5p–binding sequences—acts as a ceRNA, and decreases the levels of available miR-769-5p, thereby increasing the expression of STAT3. Consequently, the *LINC00324*–miR-769-5p–STAT3 axis enhances the malignant progression of RB cells *in vitro* and *in vivo*, thereby providing a theoretical basis for identifying novel therapeutic strategies against RB.

There are two limitations in our study. First, we demonstrated that the cancer-promoting actions of *LINC00324* in RB cells were possibly mediated by the miR-769-5p–STAT3 axis. However, miR-769-5p–STAT3 axis may not be the only target for LINC00324. We will explore the other possible mechanism events behind the oncogenic activities of *LINC00324* in RB cells. Second, one miRNA can directly target multiple mRNAs; accordingly, STAT3 may not be the only target of miR-769-5p. In our study, we only identified STAT3 as a direct target of miR-769-5p. In the near future, we will examine whether there's another possible downstream target of miR769 or if there's another player which is equally important as miR-769-5p for regulating STAT3.

In summary, *LINC00324* is upregulated in RB tumors and cell lines. The overexpression of *LINC00324* is significantly associated with worse clinical parameters and poor prognosis in patients with RB. Silencing of *LINC00324* expression reduced the aggressive behavior of RB cells *in vitro* and *in vivo* through reduced sponging of miR-769-5p and consequent downregulation of its direct target gene, *STAT3*. Our findings suggest that the *LINC00324*–miR-769-5p–STAT3 pathway is involved in the progression of RB, and that targeting this pathway may be an effective strategy for treating patients with this disease.

## MATERIALS AND METHODS

### Patients and tissue samples

This study was conducted in accordance with the principles of the Declaration of Helsinki and with the approval of the Ethics Committee of the First Affiliated Hospital of Zhengzhou University. In addition, written informed consent was obtained from all the patients participating in the study. RB tissue samples were obtained from 47 patients with RB at the First Affiliated Hospital of Zhengzhou University. Thirteen normal retinal tissue samples were collected from patients with ophthalmorrhexis treated with enucleation at the First Affiliated Hospital of Zhengzhou University. None of the patients had undergone laser photocoagulation, chemotherapy, or radiotherapy prior to the surgical procedure. All the tissue samples were instantly frozen in liquid nitrogen after surgical resection, and were preserved at −80°C until further use.

### Cell lines

Three RB cell lines (Y79, SO-RB50, and WERI-RB-1) and a normal retinal pigmented epithelial cell line, ARPE-19, were purchased from the American Type Culture Collection (Manassas, VA, USA). Dulbecco’s modified Eagle’s medium (DMEM; Gibco, Thermo Fisher Scientific, Waltham, MA, USA) supplemented with 10% of fetal bovine serum (FBS; Gibco, Thermo Fisher Scientific), 100 U/ml penicillin, and 100 mg/ml streptomycin (Gibco, Thermo Fisher Scientific) was used to culture all three RB cell lines. ARPE-19 cells were grown in Dulbecco’s modified Eagle’s medium/Nutrient Mixture F-12 (Gibco, Thermo Fisher Scientific) that was supplemented with 10% of FBS, 100 U/ml penicillin, and 100 mg/ml streptomycin. All the cells were maintained at 37°C in a humidified atmosphere containing 5% of CO_2_.

### Transfection procedures

The siRNA designed to silence *LINC00324* expression (si-LINC00324) and its negative control nonsensical sequence (si-NC) were acquired from RiboBio (Guangzhou, China). Agomir-769-5p, agomir-NC, miR-769-5p antagomir (antagomir-769-5p), and NC antagomir (antagomir-NC) were chemically synthesized by GenePharma (Shanghai, China). The pcDNA3.1 plasmid carrying the full-length *HDAC9* sequence but lacking the 3′-UTR was synthesized by GeneCopoeia (Guangzhou, China). The aforementioned molecular products were transfected into RB cells with Lipofectamine 2000 (Invitrogen, Thermo Fisher Scientific) and were subjected to the following assays.

### RT-qPCR

Total RNA was isolated using a miRNeasy Mini Kit (cat. No. 217004, Qiagen, Hilden, Germany). The purity and concentration of the extracted total RNA were evaluated by ultraviolet spectrophotometry (Nanodrop ND2000; Thermo Fisher Scientific). Reverse transcription was performed using the miScript Reverse Transcription Kit (Qiagen). Then, the expression of miR-769-5p was analyzed using a miScript SYBR Green PCR Kit (Qiagen). Small nuclear RNA U6 served as an endogenous control in the miR-769-5p expression analysis.

For quantification of *LINC00324* and *STAT3* mRNA expression, total RNA was reverse-transcribed into cDNA using a PrimeScript RT-Reagent Kit (Takara Bio, Kusatsu, Japan). The generated cDNA was subjected to qPCR using the SYBR Premix Ex Taq™ Kit (Takara Bio), with *GAPDH* as an internal reference. The 2^−ΔΔCt^ method was utilized to calculate relative gene expression.

### CCK-8 assay

At 24 h post-transfection, cells were harvested with 0.25% trypsin (1×; Gibco, Thermo Fisher Scientific), resuspended, and seeded in 96-well plates at an initial density of 2 × 10^3^ cells per well. Prior to incubation at 37°C for an additional 2 h, 10 μl of the CCK-8 reagent was added into each well. The absorbance of each well was measured at a 450 nm wavelength on an enzyme-linked immunosorbent assay microplate reader (Bio-Rad Laboratories, Inc., Hercules, CA, USA). The CCK-8 assay was performed every 24 h until 72 h after the inoculation, and the growth curve of the cells was plotted accordingly.

### Colony formation assay

For this assay, 2.5 ml of a suspension containing 1000 transfected cells was seeded in 6-well plates. The cells were maintained at 37°C and 5% CO_2,_ and cultured for two weeks. The culture medium was refreshed at three-day intervals during this period. At the end of this assay, visible colonies (> 50 cells) were fixed with 4% polyformaldehyde and stained with methyl violet. After extensive washing, the colonies were imaged and manually counted under an inverted microscope (Olympus, Tokyo, Japan).

### Apoptosis and cell cycle analyses by flow cytometry

At 48 h after transfection, cells were detached using 0.25% trypsin, washed twice with cold phosphate-buffered saline, and then used for determination of apoptosis using an Annexin V–Fluorescein Isothiocyanate (FITC) Apoptosis Detection Kit (Biolegend, San Diego, CA, USA). In brief, the cells were resuspended in 100 μl of binding buffer. After the addition of 5 μl of Annexin V–FITC and 5 μl of a propidium iodide solution, the cell suspension was incubated at room temperature in the dark for 15 min. Finally, the apoptosis rate was determined by flow cytometry (FACScan; BD Biosciences, Franklin Lakes, NJ, USA).

After 48 h culture, transected cells were collected by means of trypsin without EDTA, and subjected to the test of cell cycle status. Subsequent to extensive washing with ice-cold PBS, the transfected cells were fixed in 70% ethanol, centrifuged and then incubated with 100 μg/mL RNase at room temperature for 20 min. Thereafter, the transfected cells were stained with propidium iodide solution, after which was analyzed with flow cytometry.

### Transwell migration and invasion assays

The invasion capacity of transfected cells was tested by means of 24-well Transwell insert chambers (8 μm pores; BD Biosciences) that were precoated with Matrigel (BD Biosciences). After a 48 h incubation, the transfected cells were resuspended in 200 μl of FBS-free DMEM and inoculated into the upper chambers, and the lower chambers were filled with 600 μl of DMEM supplemented with 10% of FBS. After 24 h of incubation at 37°C in 5% CO_2_, the nontraversing cells remaining on the upper surface of the inserts were gently wiped off with a cotton swab. The invasive cells were fixed in 4% polyformaldehyde, stained with 0.1% crystal violet, and imaged. The invasive cells in five random visual fields were counted under an inverted microscope (Olympus), and these numbers were regarded as a metric of invasive capacity. The only difference between the migration assay and invasion assay was that the chambers in the migration assay were not precoated with Matrigel.

### Tumor xenograft model

The experimental procedures involving animals were approved by the Animal Ethics Committee of the First Affiliated Hospital of Zhengzhou University and were performed in accordance with the Animal Protection Law of the People’s Republic of China-2009 for experimental animals.

Short hairpin RNA (shRNA) targeting *LINC00324* (sh-LINC00324) and negative control (sh-NC) was designed and synthesized by GenePharma and used for lentivirus production. Y79 cells were transfected with lentivirus expressing sh-LINC00324 or sh-NC, and the stably transfected cells were selected using puromycin. At 5–6 weeks of age, female BALB/c nude mice were acquired from the Laboratory Animal Center of Jilin University (Changchun, China). Suspension of Y79 cells stably expressing sh-LINC00324 or sh-NC (1 × 107 cells) were collected and subcutaneously implanted into the flanks of nude mice. Each group contained three nude mice. The width and length of the tumor xenografts were measured weekly by means of a Vernier caliper, and to calculate the tumor volume, the following formula was used: tumor volume = (length × width^2^)/2. At four weeks after the injection, all the mice were euthanized by CO_2_ asphyxiation. The tumor xenografts were isolated, weighed, and analyzed by RT-qPCR and western blotting.

### Subcellular fractionation and RNA isolation

A PARIS Kit (Invitrogen; Thermo Fisher Scientific) was employed to separate the cytoplasmic and nuclear fractions of Y79 and WERI-RB-1 cells. The RNA was then extracted from the cytoplasmic and nuclear fractions using a miRNeasy Mini Kit and then subjected to RT-qPCR analysis. U6 small nuclear RNA and glyceraldehyde-3-phosphate dehydrogenase (GAPDH) were used as the nuclear and cytoplasmic controls, respectively.

### RIP assay

This assay was carried out using a Magna RIP RNA-Binding Protein Immunoprecipitation Kit (Millipore Inc., Billerica, MA, USA). In brief, cells were lysed with RIP lysis buffer, and the cell lysate was centrifuged at 12,000 × *g* for 30 min to obtain the supernatant. After that, the supernatant was incubated with magnetic beads conjugated with the anti-AGO2 or anti-IgG antibody (Millipore) at 4°C overnight, followed by digestion of the protein using Proteinase K buffer and isolation of the immunoprecipitated RNA. Relative expression was evaluated by RT-qPCR as described above.

### RNA pull-down assay

RNA pull-down assay was carried out to further determine the endogenous interaction between *LINC00324* and miR-769-5p in RB cells. First, a Pierce™ Biotin 3' End DNA Labeling Kit (Thermo Fisher Scientific, Inc.) was applied to prepare the biotinylated RNA. After this, Y79 and WERI-RB-1 cells were transfected with biotinylated miR-769-5p mimics (bio-miR-769-5p) or biotinylated negative control miRNA mimics (bio-miR-NC) by means of Lipofectamine 2000. Cells were lysed using ice-cold lysis buffer after 48 h incubation. The supernatant was harvested and subjected to addition 2 h incubation at 4°C with Dynabeads M-280 Streptavidin (BD Biosciences) to absorb bio-miR-769-5p, forming bio-miRNA-lncRNA complexes. Finally, the complexes were collected and the relative enrichment of *LINC00324* was determined in bio-miRNA-lncRNA complexes using RT-qPCR analysis.

### Bioinformatic prediction

The potential *LINC00324*–miRNA interaction was predicted using starBase 3.0 (http://starbase.sysu.edu.cn/). Two online microRNA target prediction tools, TargetScan (http://www.targetscan.org) and starBase 3.0, were utilized to search for the putative targets of miR-769-5p.

### Luciferase reporter assay

The partial 3′-UTR sequence of *STAT3* containing either the wild-type (wt) or mutant (mut) miR-769-5p–binding site was amplified by GenePharma, and subsequently inserted into a psiCHECK™-2 luciferase reporter vector (Promega Corporation, Madison, WI, USA) to construct the reporter plasmids STAT3-wt and STAT3-mut, respectively. The plasmids *LINC00324*-wt and *LINC00324*-mut were generated in the same way. For the reporter assay, either the wt or mut reporter plasmid construct along with either agomir-769-5p or agomir-NC was transfected into cells using Lipofectamine 2000. The transfected cells were collected and lysed at 48 h post-transfection, and the luciferase activity was detected via a Dual-Luciferase Reporter Assay System (Promega Corporation). The firefly luciferase activity was normalized to that of *Renilla* luciferase.

### Western blot analysis

An EpiQuik Total Histone Extraction Kit (EpiGentek, Farmingdale, NY, USA) was utilized to isolate total protein from tissues or cells. The quantification of total protein was carried out using a Bicinchoninic Acid Assay Kit (Pierce; Thermo Fisher Scientific). Equivalent amounts of proteins were separated by SDS-PAGE (10% gels) and transferred onto polyvinylidene difluoride (PVDF) membranes (Bio-Rad Laboratories). The membranes were blocked at room temperature for 2 h with Tris-buffered saline containing 0.1% of Tween 20 (TBST) that was supplemented with 5% of nonfat milk. The membranes were then incubated with anti-STAT3 antibody (cat. No. sc-8019; 1:1 000 dilution; Santa Cruz Biotechnology, CA, USA) or anti-GAPDH antibody (cat. No. sc-69778; 1:1 000 dilution; Santa Cruz Biotechnology) at 4°C overnight. The next morning, the membranes were extensively washed with TBST and then probed with a horseradish peroxidase–conjugated anti-IgG secondary antibody (cat. No. sc-516102; 1:5 000 dilution; Santa Cruz Biotechnology) for 2 h at room temperature. After three washes with TBST, an Amersham ECL Western Blotting Detection Kit (GE Healthcare Life Sciences) was used to visualize the protein signals after 1 min. Densitometric analysis was conducted using Quantity One v4.62 software (Bio-Rad Laboratories).

### Statistical analysis

All the experiments were repeated at least in triplicate, and the data were presented as mean ± standard deviation. The association between *LINC00324* levels and the clinical parameters was examined by the χ^2^ test. Student’s *t* test was applied to assess the differences between the two groups, and comparisons among multiple groups were carried out by one-way analysis of variance (ANOVA) with the Bonferroni *post hoc* test. Spearman’s correlation analysis was conducted to investigate the expression correlation between two genes. The Kaplan–Meier method and log rank test were used to evaluate the correlation between overall survival and *LINC00324* expression among the patients with RB. All the statistical analyses were conducted using SPSS software (version 18.0, SPSS, Inc., Chicago, IL, USA), and data with a *P* value < 0.05 were assumed to be significant.
